# Electrostatically driven resonance energy transfer in “cationic” biocompatible indium phosphide quantum dots†
†Electronic supplementary information (ESI) available: Detailed experimental methods, the synthesis and characterization of QDs, bioimaging, stability studies, control experiments, and the calculation of various parameters involved in the resonance energy transfer process *etc.* See DOI: 10.1039/c7sc00592j



**DOI:** 10.1039/c7sc00592j

**Published:** 2017-03-13

**Authors:** Gayathri Devatha, Soumendu Roy, Anish Rao, Abhik Mallick, Sudipta Basu, Pramod P. Pillai

**Affiliations:** a Department of Chemistry and Centre for Energy Science , Indian Institute of Science Education and Research (IISER) , Dr. Homi Bhabha Road , Pune 411008 , India . Email: pramod.pillai@iiserpune.ac.in

## Abstract

Indium Phosphide Quantum Dots (InP QDs) have emerged as an alternative to toxic metal ion based QDs in nanobiotechnology. The ability to generate cationic surface charge, without compromising stability and biocompatibility, is essential in realizing the full potential of InP QDs in biological applications. We have addressed this challenge by developing a place exchange protocol for the preparation of cationic InP/ZnS QDs. The quaternary ammonium group provides the much required permanent positive charge and stability to InP/ZnS QDs in biofluids. The two important properties of QDs, namely bioimaging and light induced resonance energy transfer, are successfully demonstrated in cationic InP/ZnS QDs. The low cytotoxicity and stable photoluminescence of cationic InP/ZnS QDs inside cells make them ideal candidates as optical probes for cellular imaging. An efficient resonance energy transfer (*E* ∼ 60%) is observed, under physiological conditions, between the cationic InP/ZnS QD donor and anionic dye acceptor. A large bimolecular quenching constant along with a linear Stern–Volmer plot confirms the formation of a strong ground state complex between the cationic InP/ZnS QDs and the anionic dye. Control experiments prove the role of electrostatic attraction in driving the light induced interactions, which can rightfully form the basis for future nano-bio studies between cationic InP/ZnS QDs and anionic biomolecules.

## Abstract 2

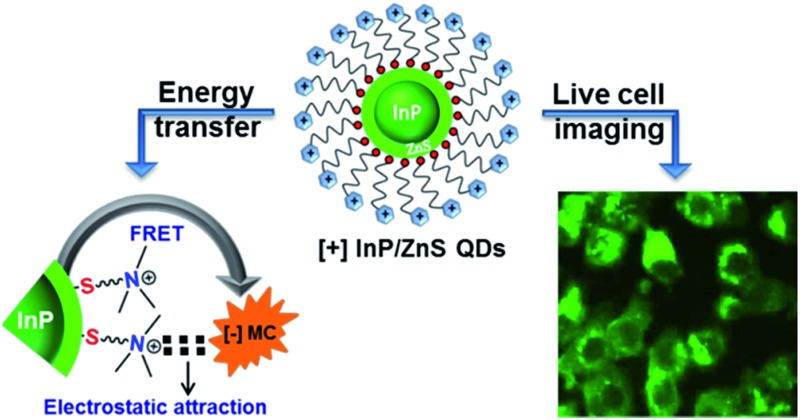
InP QDs join the family of cationic nanoparticles as a practical alternative to toxic metal ion based QDs for biological applications.

## Introduction

Electrostatic forces play a pivotal role in controlling the interactions between biomolecules and nanomaterials.^1^ In this regard, cationic nanoparticles form an integral part of nanobiotechnology as they provide complementary surface charge for binding with anionic biomolecules.^2^ The unique size dependent luminescence properties of Quantum Dots (QDs) are an added advantage over metal nanoparticles in various biomedical applications like imaging, targeting and therapeutics.^3^ One of the promising uses of QDs is in Förster Resonance Energy Transfer (FRET) based assays in monitoring various biomolecular processes including protein folding and sensing.^4–6^ To achieve this goal, extensive research has been carried out to understand the energy transfer processes in QDs based on Cd, Pb and Se, *etc.* ^4–6^ However, increasing restrictions on the use of toxic metal ions have led to the search for environmentally friendly QDs possessing adequate biocompatibility and surface chemistries. InP QDs have emerged as an alternative due to their lower toxicity and tunability of their emission in the NIR region.^7–11^ Nevertheless, the challenges associated with the synthesis of InP QDs^7b,c^ have led to limited studies on their surface engineering,^10*c*^ especially those with a cationic surface charge. In fact, there are very few reports on cationic QDs that carry a permanent positive surface charge^12,13^ in comparison to those on metal nanoparticles.^14^ The common practice is to functionalize anionic QDs with bifunctional biomolecules to impart a pH dependent cationic charge, which then facilitates bio-nano interactions.^8,15^ Among other problems, this strategy will increase the hydrodynamic diameter of the nanohybrid systems beyond the limit of renal clearance.^3*d*^ It is always advantageous to have a permanent cationic ([+]) charge on the QDs in the first place, as it reduces structural complexity. Along with surface engineering, the ability of [+] InP/ZnS QDs to participate in energy transfer needs to be explored so that the full potential of the [+] InP/ZnS QDs can be achieved. Here, we address both of the above mentioned issues and report an efficient light induced resonance energy transfer in [+] InP QDs under physiological conditions. A large bimolecular quenching constant along with a linear Stern–Volmer plot confirm the formation of a strong ground state complex between cationic InP/ZnS QDs and an anionic dye. The highlight of the present work is the use of electrostatic forces to control light induced interactions, which can form the basis for future nano-bio studies between [+] InP/ZnS QDs and [–] biomolecules. Moreover, the stable photoluminescence of [+] InP/ZnS QDs inside cells and their low cytotoxicity make them ideal candidates as optical probes for cellular imaging.

## Results and discussion

### Synthesis and characterization of [+] InP/ZnS QDs

**Scheme 1:**
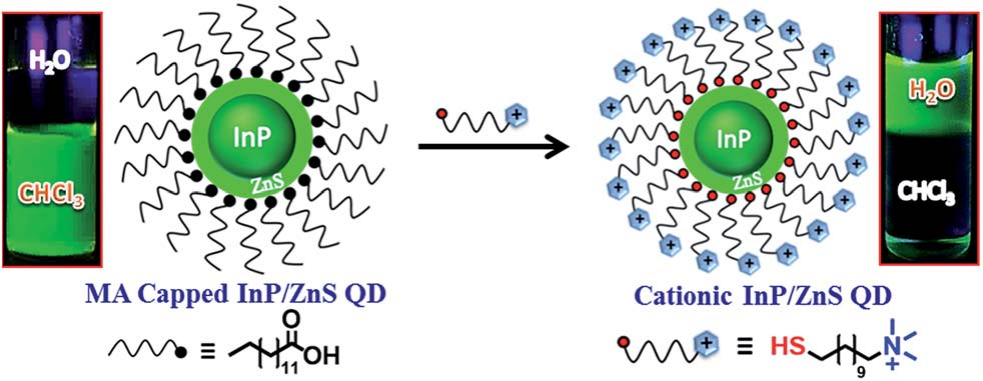
Schematics for the synthesis of [+] InP/ZnS QDs. The place exchange reaction between the myristic acid capped InP/ZnS QDs and the [+] TMA ligand is represented. The photographs of the vials show the successful transfer of [+] InP/ZnS QDs into the aqueous layer.

The InP/ZnS QDs carrying a permanent positive charge were prepared using a place exchange method (Scheme 1). The hydrophobic InP/ZnS QDs capped with myristic acid (MA), having an average core diameter of 2.8 ± 0.8 nm, were synthesized by following the reported procedures.^10b,11^ The deep electronic trap states formed by the dangling bonds on the surface of the QDs account for the broad emission of the InP/ZnS QDs (full width at half-maximum, FWHM ∼ 70 nm).^10,11^ This was further inferred from a moderate quantum yield of ∼12% for InP/ZnS QDs in chloroform, matching the reported values.^11^ The myristic acid on the surface of the InP/ZnS QDs was then replaced with the *N*,*N*,*N*-trimethyl(11-mercaptoundecyl)ammonium chloride (TMA, [+]) ligand to impart the water solubility and cationic surface charge. In a typical synthesis, 5 mL of InP/ZnS QD solution (1.5 μM) in chloroform was mixed with 2 mL of TMA solution in water (25 mg per mL). Constant stirring for ∼4 h ensured a complete phase transfer of the InP/ZnS QDs to the aqueous layer. The phase transfer process was followed by monitoring the color change of chloroform (orange to colorless) and the water layers (colorless to orange). The aqueous layer was separated and precipitated with acetone to remove excess TMA ligands, and redispersed in deionized water for further studies. The bifunctional TMA ligand helped in both the QD surface functionalization (*via* thiol group) as well as the phase transfer process (*via* the quaternary ammonium group).

**Fig. 1:**
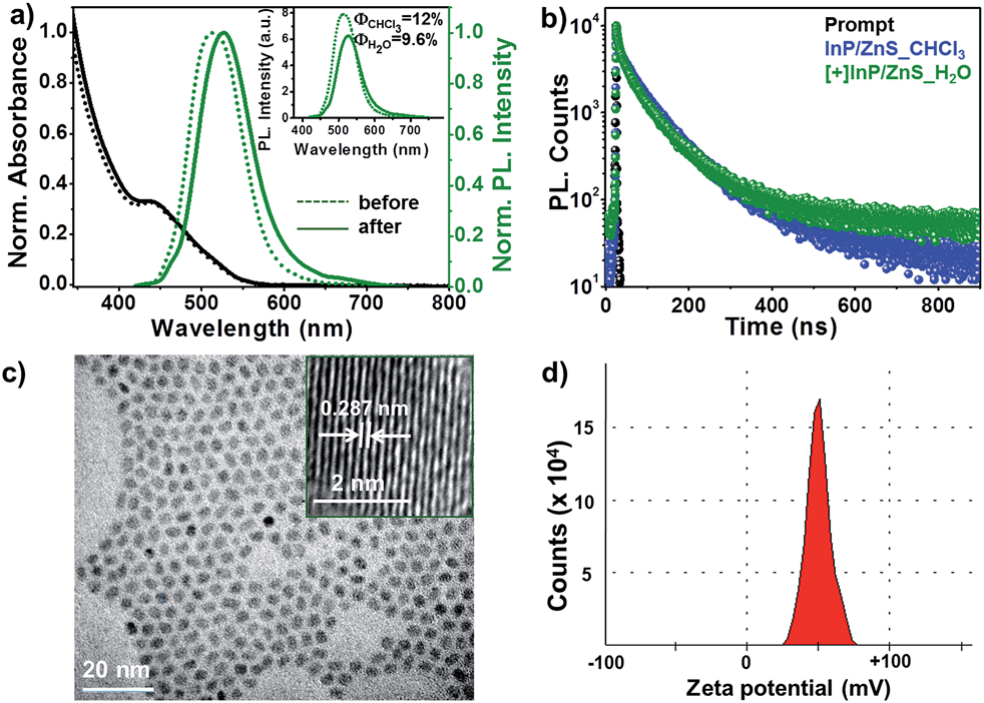
Spectroscopic and microscopic characterization of [+] InP/ZnS QDs. (a) The normalized absorption and photoluminescence spectra of InP/ZnS QDs before and after the place exchange reaction. The unnormalized photoluminescence spectra in the inset show that the [+] InP/ZnS QDs retained ∼80% of their photoluminescence after the place exchange reaction. (b) The photoluminescence decay profiles of InP/ZnS QDs before and after the place exchange reaction. (c) A representative HRTEM image of 2.8 ± 0.8 nm sized [+] InP/ZnS QDs. The inset shows the lattice fringes with an interplanar distance of 0.287 nm, corresponding to the zincblende phase of bulk InP.^17^ (d) A typical zeta potential plot (measured at pH ∼ 7) confirming the cationic charge on the surface of the InP/ZnS QDs.

The [+] InP/ZnS QDs were well characterized using spectroscopic and microscopic techniques. The steady state studies revealed a negligible change in the absorption and photoluminescence properties of the [+] InP/ZnS QDs upon place exchange (Fig. 1a and Section 2 in the ESI†). At the same time, the relative emission intensity and quantum yield calculations showed that the [+] InP/ZnS QDs retained ∼80% of their photoluminescence after place exchange (the inset of Fig. 1a and S1†). A decrease in emission is often observed in QDs upon water solubilization, which is attributed to the surface defects produced by oxidative thiol ligands.^10c,16^ The tri-exponential photoluminescence decay of the InP/ZnS QDs was retained in the cationic form with an average lifetime of ∼48 ns (Fig. 1b and S2 and Table S1†). The high resolution transmission electron microscopy (HRTEM) image shown in Fig. 1c proves the size homogeneity and crystalline nature of the [+] InP/ZnS QDs, with an interplanar distance of 0.287 nm corresponding to the zincblende phase of bulk InP.^17^ A zeta potential (*ζ*) value of +52 ± 2 mV confirmed the successful functionalization of the cationic TMA ligands on the surface of the InP/ZnS QDs. The narrow charge distribution of the *ζ* plot indicates that the [+] InP/ZnS QDs are well dispersed in the aqueous medium (Fig. 1d).

### Biocompatibility studies with [+] InP/ZnS QDs

**Fig. 2:**
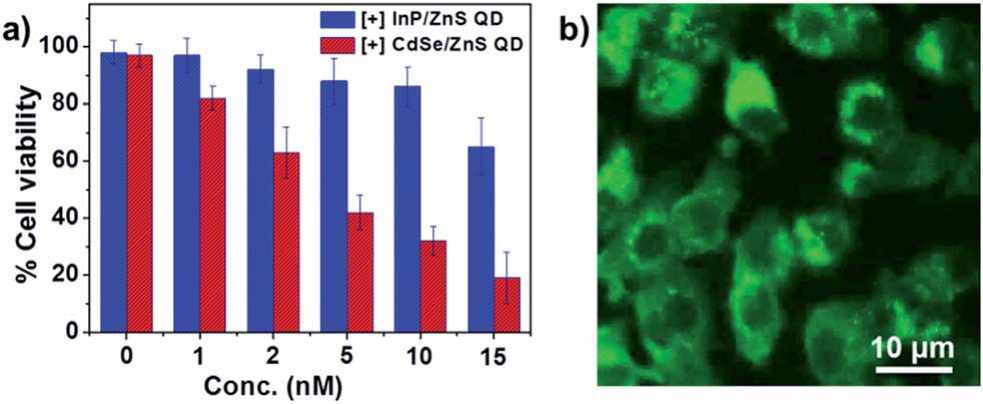
Biocompatibility of [+] InP/ZnS QDs. (a) The viability of MCF-7 cells incubated with different concentrations of [+] InP/ZnS QDs and [+] CdSe/ZnS QDs for 24 h. (b) A representative confocal image showing the fluorescence of [+] InP/ZnS QDs inside MCF-7 cells.

In order to feature in biological applications, the [+] InP/ZnS QDs should satisfy the essential prerequisites of low cytotoxicity and longtime stability in buffers and biofluids. The colloidal stability of the [+] InP/ZnS QDs was studied by monitoring their photoluminescence intensity for ∼24 h, under various physiological conditions. Fig. S5† proves that the photoluminescence, and hence stability, of the [+] InP/ZnS QDs was retained in PBS buffer and cell culture media, and in a broad range of pH values. Furthermore, the cytotoxicity of the [+] InP/ZnS QDs was tested in MCF-7 cell line using the MTT assay (the details of the cell viability studies are given in Section 3 of the ESI†). About 85% of the cells were found to be alive after ∼24 h of incubation with ∼10 nM [+] InP/ZnS QDs (Fig. 2a). The InP/ZnS QDs were found to be less cytotoxic than the [+] CdSe/ZnS QDs, having a comparable size and surface charge (the Cd^2+^ ions that were leaked from the QD core are more toxic than the In^3+^ ions, as reported previously^18^). The excellent stability and lower cytotoxicity of [+] InP/ZnS QDs paved their way as optical probes in cellular imaging studies. Live cell confocal imaging confirms the effective entry of [+] InP/ZnS QDs into MCF-7 cells (Fig. 2b and S6†). The control cellular uptake studies carried out with [–] InP/ZnS QDs showed a negligible entry of QDs inside the cells, emphasizing the role of electrostatics in the uptake mechanism (Fig. S7†). The subsistence of bright photoluminescence inside the cells, coupled with their low cytotoxicity, make [+] InP/ZnS QDs a practical alternative to toxic metal ion based QDs as optical probes for cellular imaging applications.

### Steady state resonance energy transfer studies

**Fig. 3:**
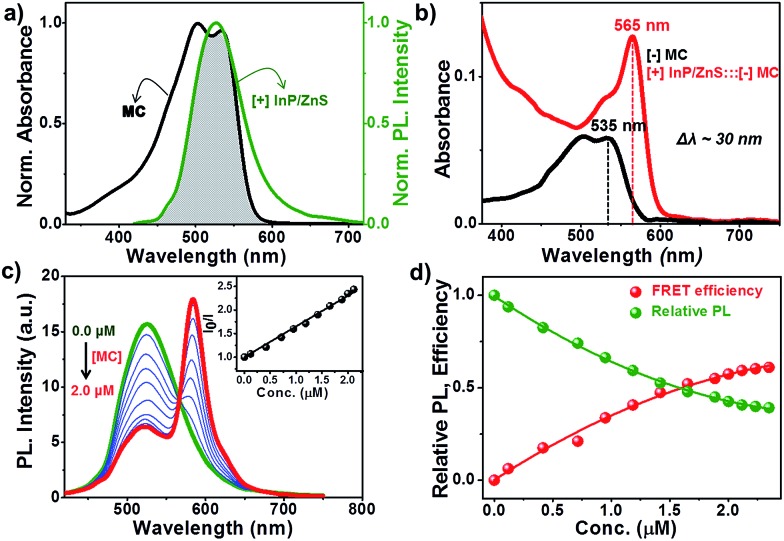
Steady state resonance energy transfer studies. (a) The spectral overlap (shaded portion) between the emission of [+] InP/ZnS QDs and the absorption of [–] MC dye. (b) A bathochromic shift of ∼30 nm was observed in the absorption of MC dye upon complexation with [+] InP/ZnS QDs. (c) Spectral changes in the emission of the [+] InP/ZnS QDs on the addition of varying concentrations of [–] MC dye. The inset is the Stern–Volmer plot showing the relative changes in the emission intensity of [+] InP/ZnS QDs as a function of [–] MC dye concentration. (d) A plot showing the saturation of the relative QD emission decay and FRET efficiency *vs.* the concentration of the MC dye.

Having established the protocol for the preparation of biocompatible [+] InP/ZnS QDs, our next focus was to utilize the cationic charge to drive the energy transfer to a complementary acceptor molecule. The idea here was to demonstrate a FRET based model study to prove the ability of [+] InP/ZnS QDs to indulge in electrostatically controlled interactions for future biological studies. In view of this, merocyanine 540 dye (MC) was selected as the acceptor due to its anionic charge and excellent water solubility (Fig. S8†).^19^ A high spectral overlap integral of 2.45 × 10^15^ M^–1^ cm^–1^ nm^4^ assured that the [+] InP/ZnS QDs and the [–] MC dye form an appropriate donor–acceptor pair for the energy transfer studies (Fig. 3a).^20^ Fig. S9† shows the changes in the absorption of the [+] InP/ZnS QDs at varying concentrations of [–] MC dye. An appreciable bathochromic shift of ∼30 nm was observed in the absorption of the MC dyes in the presence of [+] InP/ZnS QDs (Fig. 3b). The steady state emission studies were performed by selectively exciting the [+] InP/ZnS QDs at 400 nm, wherein the acceptor absorption was minimal. A gradual decrease in the [+] InP/ZnS QD emission was observed upon the successive addition of [–] MC dye, with concomitant formation of a new band corresponding to the emission of MC dye (Fig. 3c). The quenching of the [+] InP/ZnS QD emission was saturated after the addition of ∼2 μM MC dye (Fig. 3d and S10†). The possibility of direct excitation of the MC dye was ruled out by performing a control experiment wherein the dye sample (∼2 μM) was excited at 400 nm (Fig. S11†). The emission of the MC dye was red shifted by ∼13 nm in the [+] InP/ZnS:::[–] MC complex (Fig. S11†). The red shifts in both the absorption and the emission of the dye in the presence of [+] InP/Zn QDs indicate a strong ground state interaction between the QDs and the dye.^4d,11^ This was confirmed by estimating the bimolecular quenching constant by combining the slope obtained from the Stern–Volmer analysis and the lifetime of the donor (see Section 4 in the ESI† for details). The linear behavior of the Stern–Volmer plot (slope = 6.52 × 10^5^ M^–1^, the inset of Fig. 3c) and the large bimolecular quenching constant of 1.36 × 10^13^ M^–1^ s^–1^ proved that the interaction is predominantly static in nature.^20^ The electrostatic attraction between the complementary charges on the [+] InP/ZnS QDs and [–] MC dye is responsible for the strong ground state interaction observed in the [+] InP/ZnS:::[–] MC complex. The efficiency of the energy transfer process was estimated to be ∼60%, which was saturated after the addition of ∼2 μM acceptor (Fig. 3d).

### Time resolved resonance energy transfer studies

**Fig. 4:**
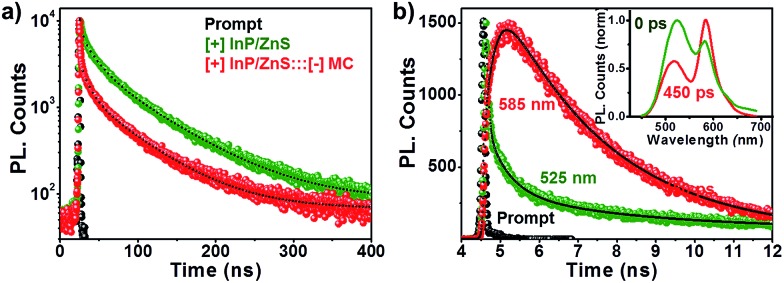
Time resolved energy transfer studies. (a) The photoluminescence decay profiles of [+] InP/ZnS QDs in the absence and presence of 2 μM [–] MC dye. (b) The photoluminescence decay profiles of the [+] InP/ZnS:::[–] MC complex collected at the emission maxima of the donor (525 nm) and acceptor (585 nm), on a 50 ns time scale. The inset shows the TRES of the [+] InP/ZnS:::[–] MC complex recorded immediately and after a time delay of 450 ps.

The process of resonance (nonradiative) energy transfer was followed using time-resolved studies. The reduction in the average lifetime of the [+] InP/ZnS QDs from ∼48 ns to ∼17 ns, in the presence of MC dyes, confirms a resonance energy transfer process in the [+] InP/ZnS:::[–] MC complex (Fig. 4a and S12 and Table S2†).^11,20^ An efficiency of ∼62% was estimated from the lifetime quenching studies, which is in close agreement with the steady state quenching results. Also, the rate of energy transfer from the [+] InP/ZnS QDs to the [–] MC dye was estimated to be 3.02 × 10^7^ s^–1^. The various parameters involved in the resonance energy transfer process between the [+] InP/ZnS QD donor and the [–] MC dye acceptor are summarized in Table S3 in the ESI.† The ultimate proof for the energy transfer process was obtained by observing the concomitant formation of the acceptor’s excited state, along with the donor decay. For this, the photoluminescence decay was collected at the emission wavelengths of both the donor and the acceptor in a shorter time scale (a time window of 50 ns). The [+] InP/ZnS:::[–] MC complex exhibited a rapid decay at ∼525 nm with a concomitant growth of the acceptor emission (Fig. 4b, S13 and Table S4 in ESI†). The presence of a negative pre-exponential factor in the fast component of the acceptor emission corresponds to the formation of an excited state of the MC dye (the growth time of the MC emission was found to be ∼250 ps).^11,20^ Finally, time-resolved emission spectroscopy (TRES) experiments were carried out to study the time dependent evolution of the emission in the [+] InP/ZnS:::[–] MC complex (the details of TRES studies are provided in the Methods section in the ESI†). The emission spectrum that was constructed immediately after the laser irradiation shows a maximum around the donor InP/ZnS QDs (inset of Fig. 4b). Interestingly, an emission maximum around the acceptor MC dye was observed when the TRES spectrum was constructed after a time delay of ∼450 ps (the inset of Fig. 4b). We can conclude from the TRES studies that the acceptor MC dye molecules are excited *via* nonradiative energy transfer from the photoexcited [+] InP/ZnS QD donor, typical of a resonance energy transfer process.^11,20^

### Proof of electrostatically driven resonance energy transfer

**Fig. 5:**
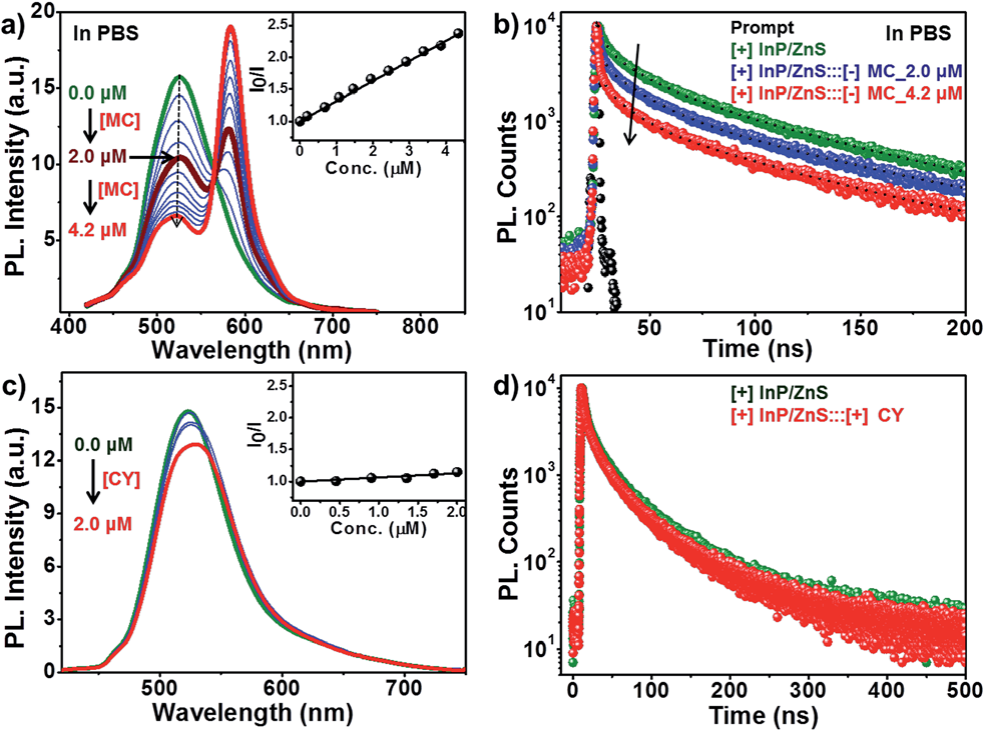
Proof of electrostatically driven resonance energy transfer. The changes in (a) the steady state and (b) the time-resolved photoluminescence of [+] InP/ZnS QDs on the addition of [–] MC dye in PBS. The inset of (a) is the Stern–Volmer plot of the [+] InP/ZnS:::[–] MC complex in PBS. (c) The steady state and (d) the time-resolved photoluminescence of [+] InP/ZnS QDs on addition of [+] CY dye in water. The inset of (c) is the Stern–Volmer plot showing negligible changes in the emission intensity of [+] InP/ZnS QDs as a function of [+] CY dye concentration.

**Scheme 2:**
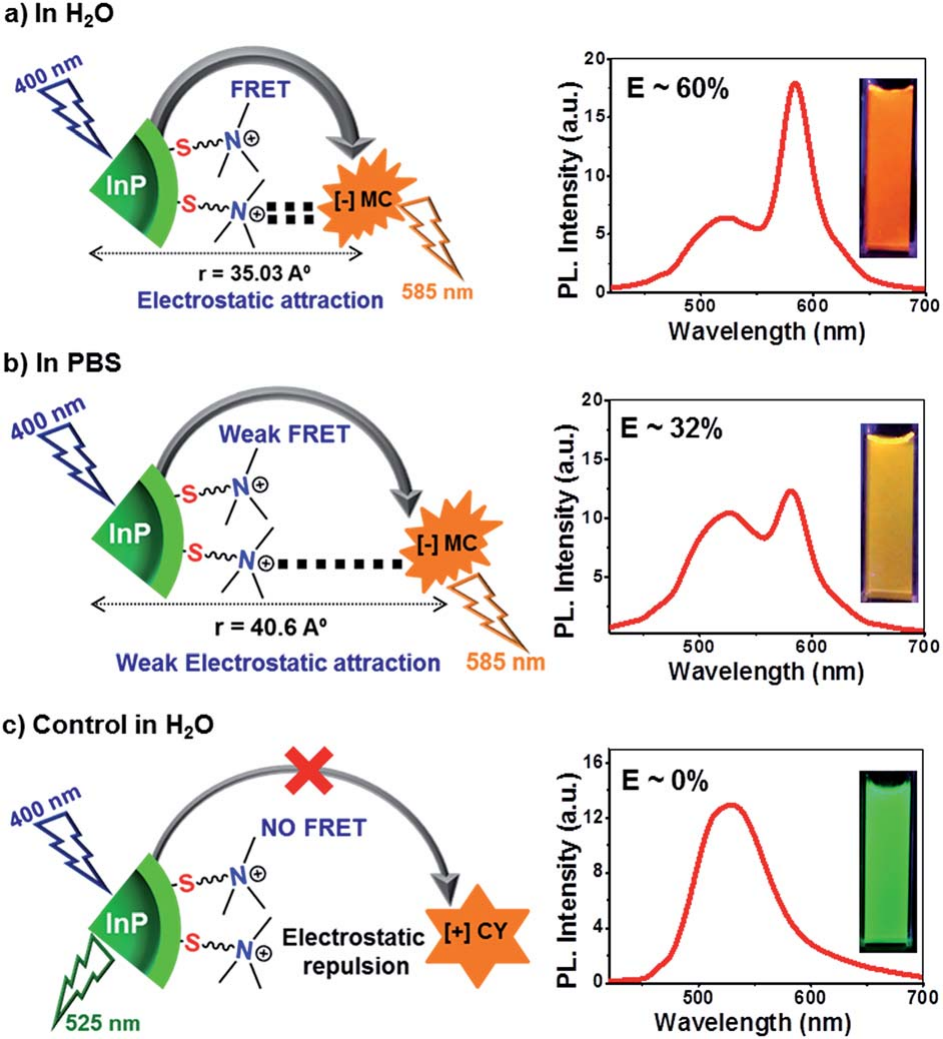
Electrostatically driven resonance energy transfer in [+] InP/ZnS QDs. (a) Efficient resonance energy transfer was observed between [+] InP/ZnS QDs and the [–] MC dye in water. (b) The presence of high concentration of salt in PBS screened the charges on the QDs and the dye, thereby decreasing the FRET efficiency. (c) No appreciable energy transfer was observed when the charges on the donor and the acceptor were the same, confirming the role of electrostatics in FRET. The steady state emission plots and optical images of the samples corresponding to each of the three conditions are shown on the right. The concentration of [+] InP/ZnS QDs and dyes was maintained at ∼0.8 μM and ∼2 μM respectively, in all of the three conditions.

The role of electrostatics in resonance energy transfer in the [+] InP/ZnS:::[–]MC complex was confirmed by performing independent control experiments. Electrostatic interactions are weakened in the presence of a high salt concentration due to the screening of the charges by the salts.^21^ Accordingly, energy transfer studies were carried out in a high salt concentration like in Phosphate Buffered Saline (PBS; Fig. 5 and S14†). An efficiency of ∼32% was obtained in PBS for the same concentration of MC dye (∼2 μM) used in water. The screening of the charges by salts weakens the electrostatic attraction between the [+] InP/ZnS QDs and the [–] MC dye, thereby lowering the efficiency. Interestingly, an efficiency similar to that observed in water was obtained when the concentration of the acceptor dye in PBS was doubled (Fig. 5a and b). Secondly, quenching experiments were performed between [+] InP/ZnS QDs and a [+] cyanine acetate (CY) dye (Fig. 5c and d, and S15†). A high spectral overlap integral of 1.56 × 10^15^ M^–1^ cm^–1^ nm^4^ suggests that the [+] InP/ZnS QDs and the [+] CY dye can form a donor–acceptor pair (Fig. S16†). However, the same charges on the surface of the donor and the acceptor prevented the formation of a complex, and no appreciable changes in both the steady state and time resolved quenching studies were observed (Fig. 5c and d). This rules out the possibility of energy transfer between the [+] InP/ZnS QDs and the [+] CY dye. Similar quenching experiments were performed with the [–] InP/ZnS QDs and the [–] MC dye, which again proved the inability of similarly charged QDs and dyes to form a stable complex (Fig. S17†). Finally, the stability studies of the [+] InP/ZnS:::[–] MC complex in biofluids revealed the breaking of the electrostatic attraction in the complex by the ions present in the medium (Fig. S18†). The dissociation of the [+] InP/ZnS:::[–] MC complex was accompanied by a reduction in the energy transfer process, and the emission of the donor InP/ZnS QDs recovered with time. All of the control experiments reiterate the role of electrostatic attraction in the formation of a strong ground state complex between the [+] InP/ZnS QDs and the [–] MC dye, leading to an efficient energy transfer process. Furthermore, the long range electrostatic field^14*a*^ helps in attracting more acceptor MC dye molecules towards the [+] InP/ZnS QD surface, thereby increasing the probability of resonance energy transfer.^22^ The schematic representation of electrostatically driven resonance energy transfer studies in [+] InP/ZnS QDs under various conditions is shown in Scheme 2.

## Conclusions

In conclusion, our work introduces InP QDs to the family of cationic nanoparticles as a practical alternative to toxic metal ion based QDs for biological applications. The two important properties of QDs, namely bioimaging and resonance energy transfer, are successfully demonstrated in [+] InP/ZnS QDs. The low cytotoxicity and stable photoluminescence of [+] InP/ZnS QDs inside cells make them ideal candidates as optical probes for cellular imaging applications. An electrostatically driven efficient resonance energy transfer was observed between [+] InP/ZnS QDs and [–] MC dye. A large bimolecular quenching constant along with a linear Stern–Volmer plot confirm the formation of a strong ground state complex between the [+] InP/ZnS QDs and the [–] MC dye. The control experiments proved the role of electrostatic attraction in driving the light induced processes, which can rightfully form the basis for future nano-bio studies between cationic InP/ZnS QDs and anionic biomolecules. The last example of the dissociation of the [+] InP/ZnS:::[–] MC complex under physiological conditions (Fig. S18†) has the potential to be carefully translated into the FRET based signalling and targeting of biomolecular processes.
