# Supplementary material for: Electrostatically driven resonance energy transfer in “cationic” biocompatible indium phosphide quantum dots
Source: Chem Sci. 2017 Mar 13;8(5):3879–84. doi: 10.1039/c7sc00592j (PMC5465571; doi:10.1039/c7sc00592j)
Supplement: Supplementary file 1 [file SC-008-C7SC00592J-s001.pdf]

Electronic Supplementary Material (ESI) for Chemical Science.
This journal is © The Royal Society of Chemistry 2017

Electronic Supplementary Information

Electrostatically driven resonance energy transfer in "cationic" biocompatible indium phosphide quantum dots

Gayathri Devatha, Soumendu Roy, Anish Rao, Abhik Mallick, Sudipta Basu, and Pramod P. Pillai*

Department of Chemistry and Centre for Energy Science, Indian Institute of Science Education and Research (IISER),
Dr. Homi Bhabha Road, Pune 411008, Maharashtra, India

*Correspondence to: pramod.pillai@iiserpune.ac.in

**Table of contents**	**Page No.**
1. Section 1: Experimental details and Methods	**S2-S7**
2. Section 2: Characterization of [+] InP/ZnS and [+] CdSe/ZnS QDs	**S8-S11**
3. Section 3: Biocompatibility and Bioimaging with [+] InP/ZnS QD	**S12-S13**
4. Section 4: Background of Förster Energy Transfer Formalism	**S14-S16**
5. Section 5: Resonance energy transfer studies	**S17-S25**
6. Section 6: Stability studies of [+] InP/ZnS::: [-] MC complex	**S26**

Section 1: Experimental Details

Materials and Methods:

Indium acetate, Myristic acid (MA), Octylamine, Cadmium Oxide (CdO), Trioctylphosphine Oxide (TOPO), Trioctylphosphine (TOP), Selenium powder (Se), Diethyl Zinc Solution (1.0 M in hexanes) ($(Et)_2Zn$), Hexamethyldisilathiane (HMDST), 1-Octadecene (ODE), Hexadecylamine (HDA), Tetramethylammonium hydroxide (TMAOH) 25 % wt. in water, 11-mercaptoundecanoic acid (MUA), Merocyanine-540 (MC) and 1, 1'-diethyl- 2, 2'-cyanine iodide (CY) were purchased from Sigma-Aldrich. Tris (trimethylsilyl phosphine) was purchased from Strem chemicals. Dodecyl Amine (DDA), 1-Tetradecylphosphonic acid (TDPA), were purchased from Alfa Aesar. DMEM media and 3-(4, 5-dimethylthiazol-2-yl)-2,5-diphenyltetrazolium bromide (MTT) were purchased from HiMedia. All the reagents were used as received without any further purification. N, N, N-Trimethyl(11-mercaptoundecyl)ammonium Chloride (TMA) was synthesized according to reported procedure.[1]

Synthesis of InP/ZnS quantum dots:

The synthesis was carried out by following reported procedures.[2,3] Briefly, Indium acetate (0.4 mmol, 0.112 g), Myristic acid (1.54 mmol, 0.35g) and 5 ml Octadecene were loaded in a three-necked RB and heated to 120°C under N_2 atmosphere. The reaction mixture was evacuated for 30 min at 120°C, further raised to 200°C and a solution containing tris(trimethylsilyl phosphine) (0.2 mmol, 58 μL) and octylamine (2.4 mmol, 400 μL) in octadecene (1 mL) was injected. After desired crystal growth, the reaction was arrested by reducing the reaction temperature to 130°C. The InP QDs formed were further overcoated with ZnS in the same reaction vessel. An injection mixture containing diethylzinc (684 μL, 1M solution in hexane) and hexamethyldisilathiane (128 μL) in 2 ml octadecene was added

dropwise, under gentle stirring over a period of 30 min. After the addition is complete, the reaction mixture was raised to 200°C and stirring was continued for 2 h, resulting in the formation of core shell InP/ZnS QDs. The reaction mixture was then cooled to room temperature by adding 5 ml of cold Toluene to arrest the growth of ZnS shell. The resultant InP/ZnS QDs were purified by precipitating with ethanol and redispersing in chloroform. This step was repeated two times and the purified QDs were dispersed in chloroform for further studies.

Synthesis of TOPO capped CdSe QDs:

The synthesis was carried out by following the reported procedure.[4] Briefly, CdO (0.35 g, 0.26 mmol), Dodecyl Amine (3 ml), Tetradecylphosphonic acid (0.21 g, 0.72 mmol), Trioctyl phosphine Oxide (1.35 g, 3.45 mmol) were loaded in a 50 ml of three-necked RB and heated to 100 ^{0}C under N_2 atmosphere. The temperature of reaction mixture was further increased to 300 ^{0}C to form a clear solution. The solution containing Se (0.032 g, 0.26 mmol) in 1 ml TOP was injected to the hot solution. After desired crystal growth, the reaction was arrested by reducing the reaction temperature to ambient conditions and the QDs thus obtained were purified.

Synthesis of TOPO capped CdSe/ZnS QDs:

A reaction mixture containing HDA, octadecene, TOPO capped CdSe nanoparticles were heated to 100 ^{0} C in an inert atmosphere for 30 min. The mixture was further heated up to 150 ^{0}C, and an injection mixture containing calculated amount of $(Et)_2Zn$ and HMDST in 2 ml of TOP was added dropwise, under gentle stirring. The temperature was further raised to 180 ^{0}C for 30 min. Then the reaction mixture was cooled to room temperature and the resultant QDs were purified by precipitating with methanol, followed by centrifugation (3 times) and redispersed in chloroform.

Preparation of water-soluble [+] QDs:

The water-soluble [+] InP/ZnS and [+] CdSe/ZnS QDs were prepared through a place exchange reaction. In a typical place exchange experiment, 5 ml of QDs solution in chloroform (1.5 μM) was mixed with 2 ml of TMA solution (25 mg per mL). The mixture was stirred for about 4 h leading to a complete phase transfer of QDs from the organic phase to the aqueous phase. The phase transfer can be easily monitored by the color change of chloroform (orange to colorless) and water (colorless to orange) phases. The bifunctional TMA ligand helped in both QD surface functionalization (via thiol group) as well as in the phase transfer process (via quaternary ammonium group). The aqueous phase was carefully separated and precipitated with acetone to remove excess TMA ligands. This step was repeated two times. Finally, the cationic QDs were redispersed in deionized water for further studies. A similar procedure was adopted for the preparation of anionic [-] InP/ZnS QD by feeding a basic solution of 11-mercapto undecanoic acid as the ligand during the place exchange reaction.

Instrumentation:

UV-Vis absorption studies: UV-Vis absorption studies were carried out on Shimadzu UV-3600 UV/Vis/NIR spectrophotometer in an optical quartz cuvette (10 mm path length) over the entire range of 400 – 800 nm. Concentration of the [+] InP/ZnS QD was taken in such a way that the optical density of the solution was ~ 0.1 at excitation wavelength 400 nm. This corresponds to ~ 0.8 μM of [+] InP/ZnS QD. All the photophysical studies were carried out with 3 mL of ~ 0.8 μM of [+] InP/ZnS QD.

Photoluminescence studies:

The photoluminescence studies were performed on a Fluorolog-3 spectrofluorometer (HORIBA Scientific). The energy transfer studies were performed by exciting at 400 nm where the acceptor dyes (MC and CY) have minimal absorption.

Energy transfer experiments:

Energy transfer experiments were performed with different charged InP/ZnS QDs donors and dye acceptors. In a typical experiment, a 3 mL aqueous solution of charged InP/ZnS QDs was prepared such that the absorbance was ~ 0.1 at the excitation wavelength (400 nm, corresponding to a concentration of ~0.8 μM). Different aliquots of oppositely charged dyes were added sequentially to the QD solution and spectral changes were monitored by using Shimadzu UV-3600 and Fluorolog-3 (HORIBA Scientific) spectrofluorometers. The corresponding lifetime measurements were carried out in an IBH picosecond time correlated single photon counting (TCSPC) system, upon excitation with a 405 nm laser source. The fluorescence decay profiles were de-convoluted using IBH data station software version 2.1, and fitted with exponential decay, minimizing the $\chi2$ values.

Time-resolved emission spectroscopic (TRES) experiment:

TRES measurement is an excellent technique to monitor the excited state dynamics of molecules and to identify multiple emissive species present in a given system. TRES experiment of [+] InP/ZnS::: MC complex was carried out by collecting the transient lifetime profiles in the spectral range of 450−690 nm, with an interval of 4 nm. Time-resolved emission spectra were constructed by slicing the transient profiles obtained at each wavelength.

High Resolution Transmission Electron microscope (HRTEM) studies:

A drop of [+] InP/ZnS solution was placed on a 400 mesh carbon-coated copper grid (Ted Pella, Inc.), at ambient conditions and allowing the excess solvent to evaporate under air in dust free conditions. The samples were further dried under vacuum and imaged on a JEOL 200 kV high-resolution transmission electron microscope.

Zeta potential measurements:

An aqueous solution of [+] InP/ZnS QD (~ 0.8 μM) was used in zeta-potential (ζ)studies by using a Zetasizer Nano series, Nano-ZS90 (Malvern Instruments, U.K.) equipped with 655 nm laser. The error was calculated from three different measurements on three different samples.

ζ was determined by measuring the electrophoretic mobility and using Henry's equation –

$$U_E = \frac{2\varepsilon z f(\kappa_a)}{3\eta}$$

Where,

U_E = electrophoretic mobility

z = zeta potential

ε = dielectric constant

η = viscosity

$f(\kappa_a)$ = Henry's function

Smoluchowski's approximation was used to measure the zeta potential values of NPs.

MTT assay:

The cytotoxicity studies were performed on MCF-7 cell line using tetrazolium salt, 3,4,5-dimethylthiazol-2,5-diphenyltetrazolium bromide (MTT) assay. MCF-7 cells were seeded in 96-well plate (in DMEM with 10% FBS, 1% penicillin and 10 μg/mL insulin) and incubated overnight in a 5% CO_2 incubator at 37 °C for attachment. After achieving a confluence of ~ 85 %, the cells were incubated with different concentrations of QDs for ~ 24 h. Then 20 μL of MTT reagent (5 mg/mL) was added to each well and incubated for 4 h at 37 °C. The purple formazan crystals formed from the reduction of MTT by mitochondrial dehydrogenase enzyme were solubilized in 100 μL of DMSO. The absorbance of formazan crystals at 570

nm was measured using a micro plate reader (Varioskan Flash) and was representative of the number of viable cells per well. The absorbance from QD was negligible in the concentration range studied. All samples were assayed in triplicate and the percent cell viability was calculated considering the untreated cells as 100% viable.

Confocal Microscopy:

Confocal imaging was performed on a Zeiss LSM 710 microscope. MCF-7 cells were seeded (5×10^4 cells/dish) on a coverslip in a 6 well plate and incubated overnight in a 5% CO_2 incubator at 37 °C. Next day, old media was removed and cells were washed with cold PBS and ~ 10 nM [+] InP/ZnS QD solution was added to the cells and incubated for 4 hrs. Thereafter, cells were washed with PBS three times before taking the images. The excitation source used was ~ 400 nm laser.

Section 2: Characterization of [+] InP/ZnS QD

Steady state emission of InP/ZnS QDs:

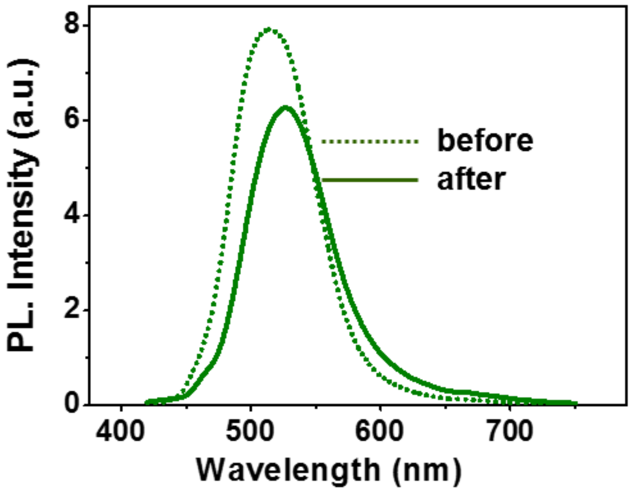

Fig. S1 Steady state photoluminescence of InP/ZnS QD before (dotted spectrum) and after (solid spectrum) place exchange with [+] TMA ligand. Approximately 80% of the photoluminescence is retained in InP/ZnS QDs after place exchange.

Calculation of photoluminescence Quantum yield:

Coumarin 153 (quantum yield ~ 0.58) was used as quantum yield reference[5] for measuring the quantum yield. The quantum yield of InP/ZnS QD was determined according to the following equation.[6]

$$\emptyset_s = \emptyset_r \left(\frac{F_s\, A_r}{A_s\, F_r}\right) \frac{\eta_s^2}{\eta_r^2}$$

Where Φ_s & Φ_r = Quantum yields of QD and reference, respectively

F_s & F_r = Integrated fluorescence intensities of QD and reference, respectively

A_s & A_r = Absorbance at the excitation wavelength of QD and reference, respectively

η_s & η_r = Refractive indices of solvents for QD and reference, respectively

The Quantum yield of InP/ZnS in $CHCl_3$ was obtained to be 0.12 (12 %). This is in accordance with the literature reports.[3]

The Quantum yield of [+] InP/ZnS in H_2O was estimated to be 0.096 (9.6 %).

Lifetime studies:

InP/ZnS QD before and after place exchange:

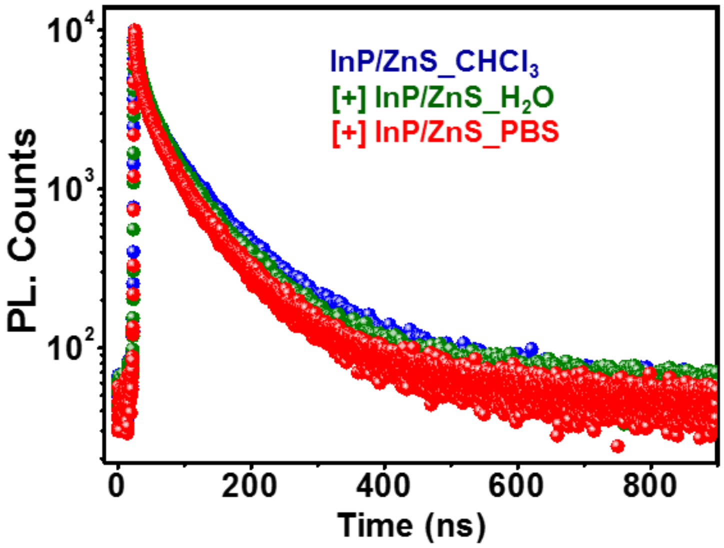

Fig. S2 Photoluminescence decay profile of InP/ZnS in $CHCl_3$ (blue decay), water (green decay), PBS (red decay) upon excitation with a 405 nm laser source.

Table S1: Photoluminescence decay analysis of InP/ZnS QD in a time window of 1μs.

Sample	τ_1 (ns)	A_1	τ_2 (ns)	A_2	τ_3 (ns)	A_3	avg τ (ns)	Chi.Sq
InP/ZnS_$CHCl_3$	1.29	0.66	43.75	0.26	112.6	0.08	71.25	1.15
[+] InP/ZnS_Water	2.98	0.49	18.2	0.35	70.23	0.16	47.69	1.11
[+] InP/ZnS_PBS	2.56	0.47	15.17	0.34	66.87	0.19	48.82	1.09

EDX analysis of [+] InP/ZnS QDs:

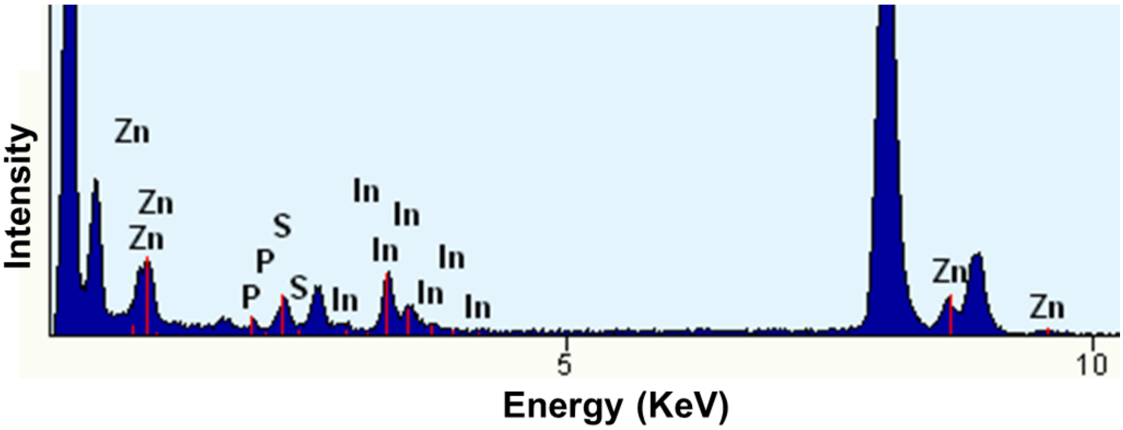

Fig. S3 EDX spectrum of [+] InP/ZnS QD studied during HRTEM analysis confirms the presence of In, P, Zn and S in the QDs.

Steady state absorption and photoluminescence of CdSe/ZnS QDs:

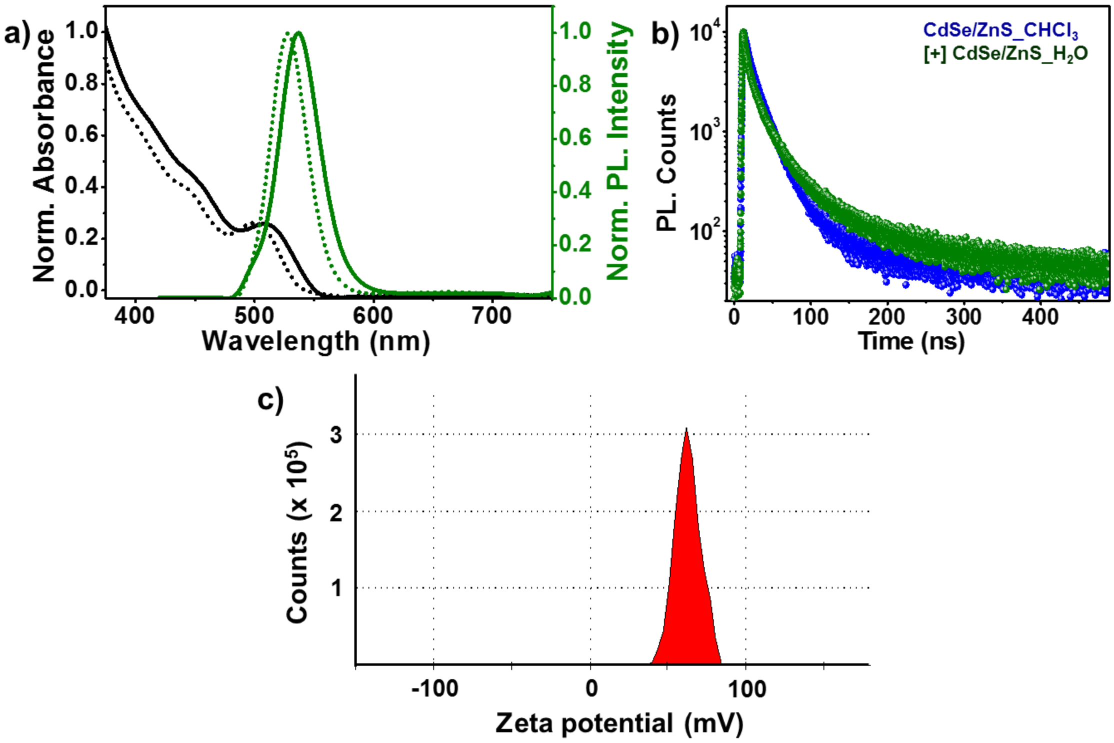

Fig. S4 a) Normalized Absorption (black spectrum) and photoluminescence (green spectrum) of CdSe/ZnS QD before (dotted spectrum) and after (solid spectrum) place exchange. **b)** Photoluminescence decay profile of CdSe/ZnS QD before (blue spectrum) and after (green spectrum) place exchange upon 405 nm excitation. b) Zeta potential plot confirming the cationic charge on the surface of CdSe/ZnS QDs. A ζ + 59.1 $\pm$ 2.7 was observed and the error was calculated from three different measurements on three different samples.

The concentrations of the QDs were determined from the size and first excitonic peak using the following equations:

Calculation of concentration of [+] InP/ZnS QD:

The concentration was estimated from the optical absorption spectra of [+] InP/ZnS QDs by using Beer lambert Law

$$\boldsymbol{A = \varepsilon C l} \qquad (1)$$

Here, A is the absorbance at first excitonic peak, ε is the molar extinction coefficient in L $mol^{-1}cm^{-1}$, C is the concentration and l is the path length of the cuvette (1cm).

ε is calculated from following equation[7]

$$\boldsymbol{\varepsilon = 3046.1(D)^3 - 76532(D)^2 + (5.5137\ X\ 10^5)(D) - (8.9839\ X\ 10^5)} \qquad (2)$$

Here, D, the diameter of the [+] InP/ZnS QD was estimated to be 2.7 nm from the following equation[7]

$$\boldsymbol{D = (-3.7707\ X\ 10^{-12})\lambda^5 + (1.0262\ X\ 10^{-8})\lambda^4 - (1.0781\ X\ 10^{-5})\lambda^3 + (5.4550\ X\ 10^{-3})\lambda^2 - (1.3122)\lambda + 119.9} \qquad (3)$$

Here, λ is the wavelength at first excitonic peak in nm (440 nm). The ε was calculated to be 92347.1 L $mol^{-1}cm^{-1}$. Concentration of 0.8 μM was obtained upon substituting the value of ε in equation (1) with OD of 0.074 at 440 nm.

Calculation of concentration of [+] CdSe/ZnS QD:

The diameter, D, of [+] CdSe/ZnS QD was estimated to be 2.5 nm from the following equation[7]

$$\boldsymbol{D = (1.6122\ X\ 10^{-9})\lambda^4 - (2.6575\ X\ 10^{-6})\lambda^3 + (1.6242\ X\ 10^{-3})\lambda^2 - (0.4277)\lambda + 41.57} \qquad (4)$$

Here, λ is the wavelength at first excitonic peak in nm (510 nm). The ε is calculated from following equation[7]

$$\boldsymbol{\varepsilon = 5857\ (D)^{2.65}} \qquad (5)$$

The ε was calculated to be 66407.4 L $mol^{-1}cm^{-1}$. The concentration of [+] CdSe/ZnS QD was estimated by substituting the ε value in (1).

Section 3: Biocompatibility and Bioimaging with [+] InP/ZnS QD

Stability of [+] InP/ZnS QD

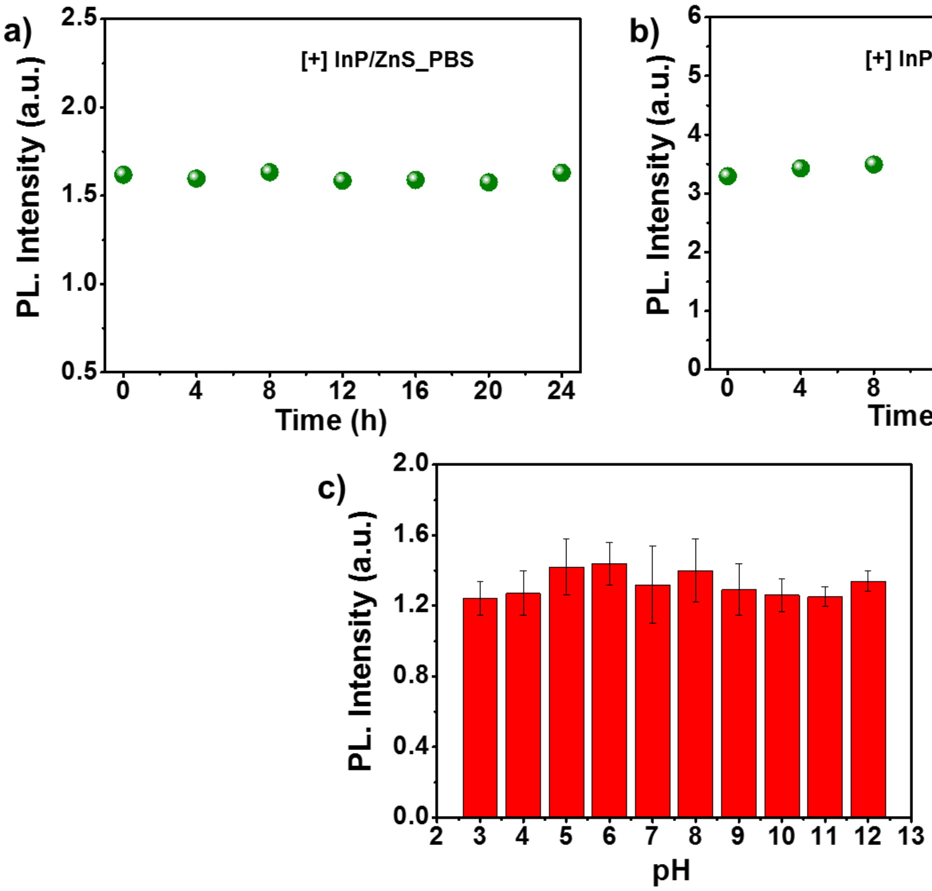

Fig. S5 The photoluminescence, and hence stability, of [+] InP/ZnS QD in a) PBS and b) DMEM media (with 10 % FBS) and c) different pH values.

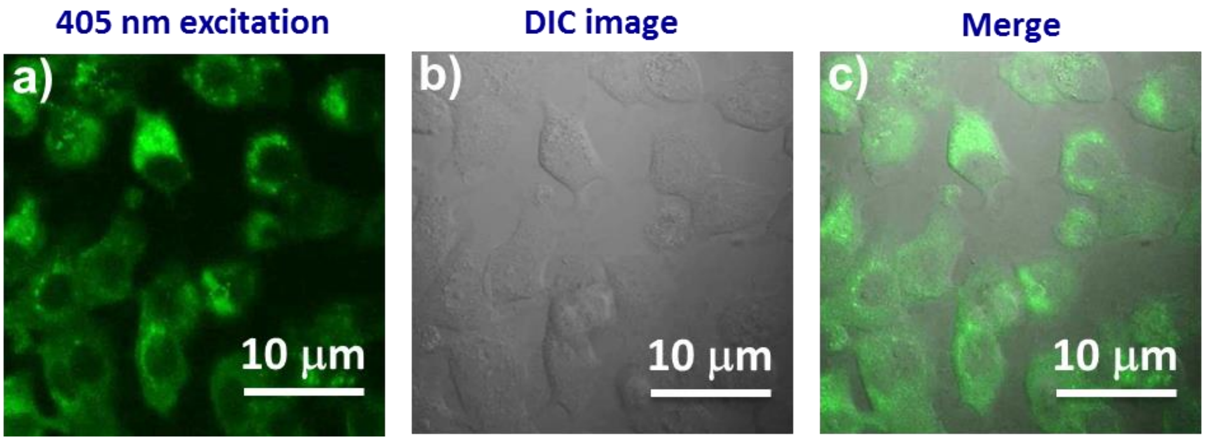

Fig. S6 Fluorescent cell imaging by confocal microscopy of [+] InP/ZnS QD (10 nM) incubated with MCF-7 cells for 4 h: a) fluorescence image upon excitation of 400 nm laser source, b) Phase contrast (DIC) image and c) Fluorescence image merged with DIC image.

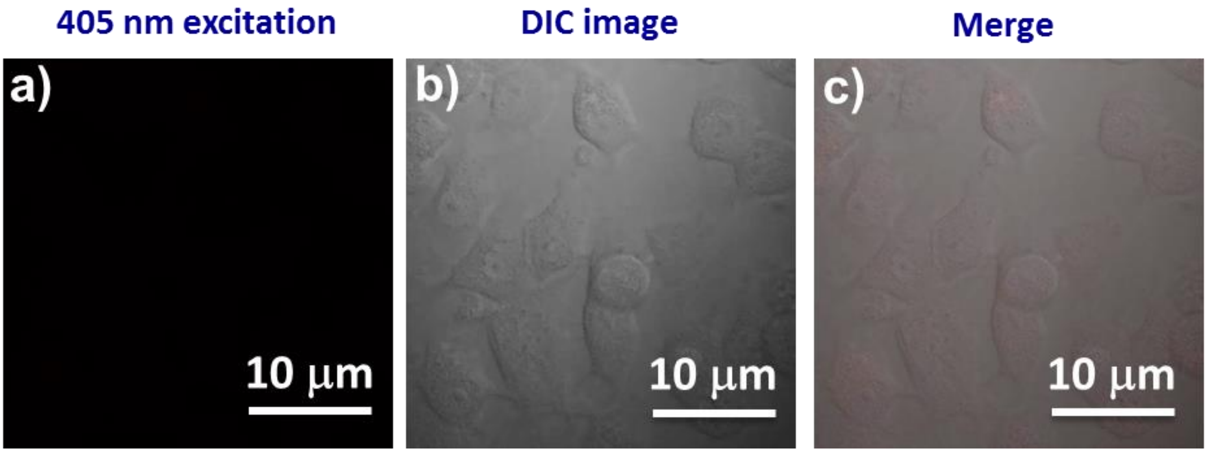

Fig. S7 Fluorescent cell imaging by confocal microscopy of [-] InP/ZnS QD (10 nM) incubated with MCF-7 cells for 6 h: a) fluorescence image upon excitation of 400 nm laser source, b) Phase contrast (DIC) image and c) Fluorescence image merged with DIC image.

Section 4: Background of Förster Energy Transfer Formalism

Förster resonance energy transfer (FRET) is a non-radiative energy transfer between excited fluorescent donor and a ground state acceptor molecule. In FRET the energy is transferred nonradiatively from donor to acceptor.[5] This process results from the dipole-dipole interactions and is thus strongly dependent on the distance between the donor and acceptor. It requires an appreciable spectral overlap between donor emission and acceptor absorption.

The rate of energy transfer for a D-A pair separated by a distance r can be expressed as

$$k_T(r) = \frac{1}{\tau_D}\left(\frac{R_0}{r}\right)^6 \qquad (6)$$

Where, R_0 is given by

$$R_0 = 0.2018 \left(\frac{\emptyset_D\, \kappa^2 J(\lambda)}{\eta^4}\right)^{1/6} A^0 \qquad (7)$$

And

$$J(\lambda) = \int_0^{\infty} F_D(\lambda)\epsilon_A(\lambda)\lambda^4\, d\lambda \quad M^{-1}\, cm^{-1}\, nm^4 \qquad (8)$$

Where, R_0 is the Förster distance at which the efficiency is 50%, r is the distance between donor and acceptor, τ_D is the lifetime of donor in absence of the acceptor, $\emptyset_D$ is the quantum yield of the donor in absence of acceptor, κ^2 is the orientation factor for the dipoles, J(λ) is the spectral overlap between the emission of donor and absorption of the acceptor, η is the refractive index of the medium. F_D (λ) Normalized fluorescence intensity of the donor at a particular wavelength, ϵ_A is the molar extinction coefficient of the acceptor.

The efficiency of FRET can be calculated from steady state emission and lifetime experiments.

From steady state emission quenching,

$$\boldsymbol{E = \frac{F_{DA}}{F_D}} \qquad \textbf{(9)}$$

From lifetime quenching studies,

$$\boldsymbol{E = \frac{\tau_{DA}}{\tau_D}} \qquad \textbf{(10)}$$

F_{DA}, τ_{DA} are the fluorescence intensity and lifetime of the donor in the presence of acceptor and F_D, τ_D are the fluorescence intensity and lifetime of the donor in the absence of acceptor.

The nature of binding between the [+] InP/ZnS QDs and [-] MC dye was examined by Stern−Volmer analysis. The Stern−Volmer quenching constant was determined by the following equation

$$\boldsymbol{\frac{I_0}{I} = 1 + K_{SV}[Q]} \qquad \textbf{(11)}$$

$$\boldsymbol{K_{SV} = k_q \tau_0} \qquad \textbf{(12)}$$

Where I_0 is the emission intensity of the donor [+] InP/ZnS QDs in the absence of the [-] MC dye, I is the emission intensity of donor in the presence of acceptor [-] MC, K_{SV} is the Stern-Volmer constant, [Q] is the concentration of the quencher [-] MC dye, k_q is the bimolecular quenching constant and τ_0 is the lifetime of donor [+] InP/ZnS QD in the absence of acceptor [-] MC dye.

The distance between donor and acceptor was calculated from the following equation:

$$\boldsymbol{E} = \frac{\boldsymbol{R_0^6}}{\boldsymbol{R_0^6} + \boldsymbol{r^6}} \qquad (\mathbf{13})$$

Here, E is the energy transfer efficiency calculated from the steady state experiments by using equation (9), R_0 is the Förster distance at which the efficiency is 50% (calculated from equations (7) and (8)) and r is the distance between donor and acceptor.

Section 5: Resonance energy transfer studies

Dye Properties:

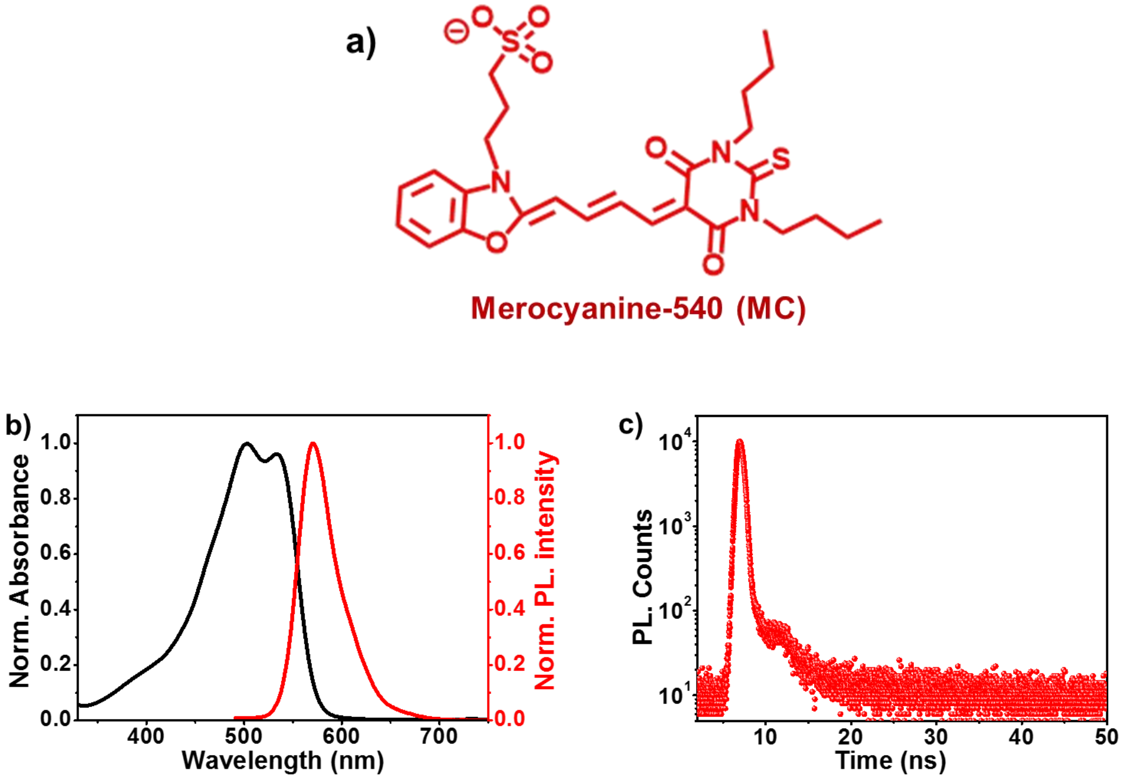

Fig. S8 a) Chemical structure of Merocyanine-540 (MC) dye. b) Normalized absorption (black curve) and photoluminescence (red curve) spectra of [-] MC dye in water. c) Photoluminescence decay profile of [-] MC dye in water upon excitation with a 405 nm laser source.

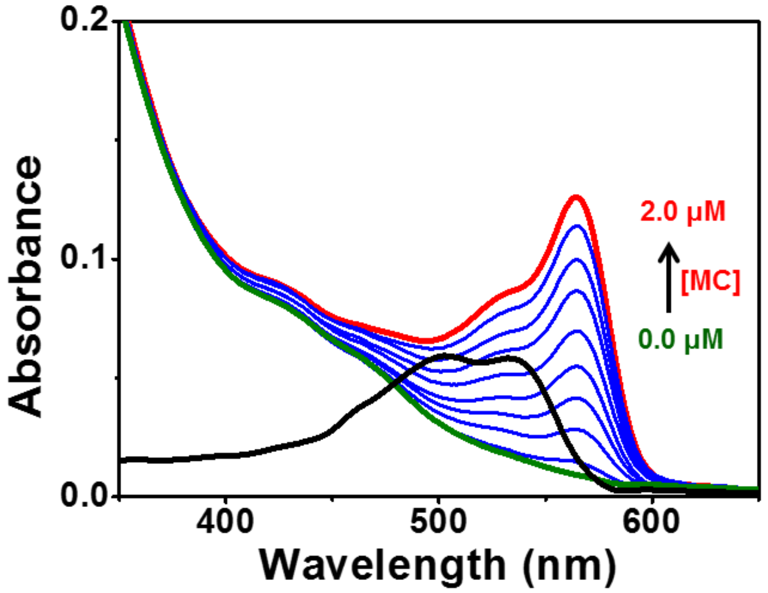

Fig. S9 Absorption spectral changes of [+] InP/ZnS QDs with increasing concentration of [-] MC dye. The absorption spectrum of [-] MC alone is shown as the black curve.

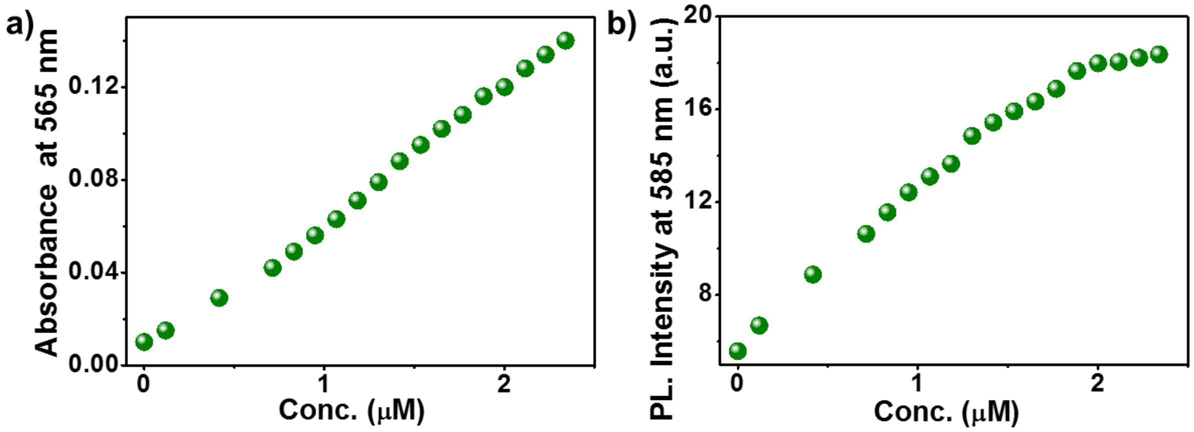

Fig. S10 Variation in a) absorption (monitored at 565 nm) and b) photoluminescence (monitored at 585 nm) intensities of [+] InP/ZnS QD:::[-] MC complex with increasing concentration of [-] MC dyes are shown. The absorption intensity keeps on increasing with the addition of [-] MC dyes to [+] InP/ZnS QDs. Interestingly, the photoluminescence intensity got saturated after ~ 2μM addition of [-] MC dyes, indicating that the MC dye is getting excited through a nonradiative energy transfer from InP/ZnS QDs.

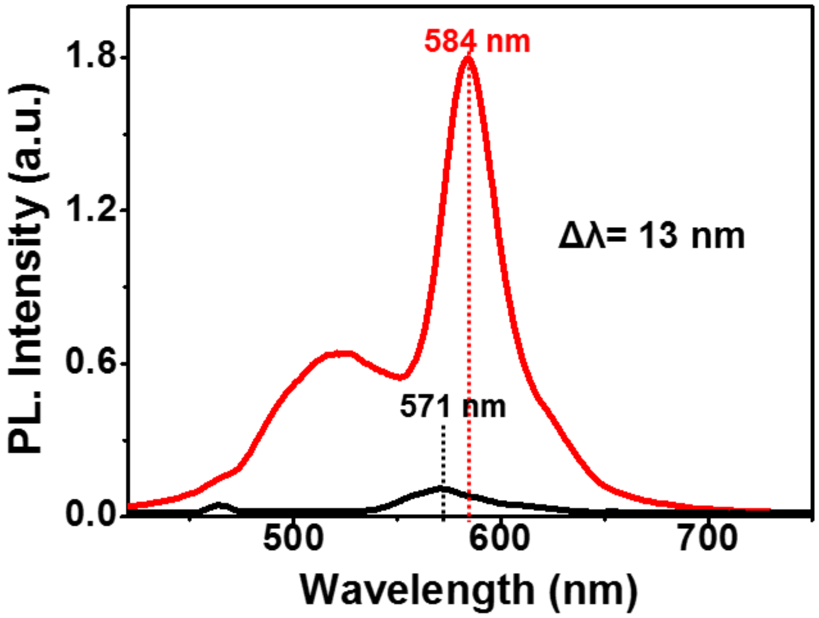

Fig. S11 Photoluminescence spectra of [-] MC in the absence (black spectrum) and presence (red spectrum) of [+] InP/ZnS QD respectively. The photoluminescence of MC dye is red shifted by ~ 13 nm in InP/ZnS QD-MC dye complex confirming a strong ground state interaction between the QD and dye

[+] InP/ZnS QD decay analysis in the presence of [-] MC dye, at longer time scale:

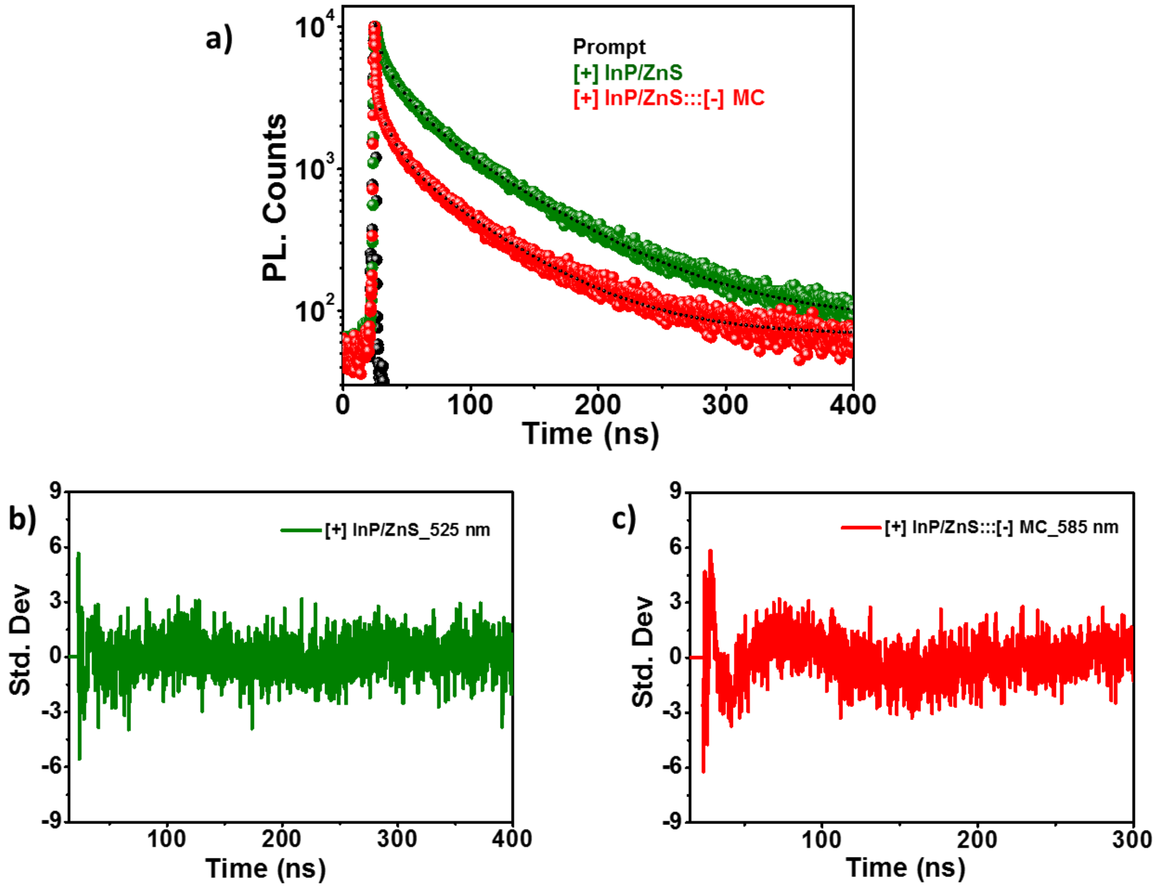

Fig. S12 a) Photoluminescence decay profile of [+] InP/ZnS in the absence (green decay) and presence (red decay) of [-] MC monitored at 525 nm, upon excitation with on 405 nm laser source. The residual plots of the fit corresponding to the decays of [+] InP/ZnS in the b) absence and c) presence of [-] MC is shown.

Table S2: Photoluminescence decay analysis of [+] InP/ZnS QD in the presence of [-] MC dye measured in a time window of 1 μs.

Sample	τ_1 (ns)	A_1	τ_2 (ns)	A_2	τ_3 (ns)	A_3	avg τ (ns)	Chi.Sq
[+] InP/ZnS_Water	2.98	0.49	18.2	0.35	70.23	0.16	47.69	1.11
[+] InP/ZnS_2.0μM _MC	0.98	0.79	11.45	0.20	60.43	0.01	17.30	1.13

Table S3: Energy transfer parameters for [+] InP/ZnS:::[-] MC system in water and PBS

Sample	J(λ) (M^{-1} cm^{-1} nm^{4})	R_0 (Å)	E^a (%)	E^b (%)	r_0 (Å)	$K_T(r)$ (S^{-1})
**[+] InP/ZnS_MC in H_2O_2.0 µM**	$2.45x10^{15}$	37.23	60.0	62.0	35.03	3.02×10^{7}
**[+] InP/ZnS_MC in PBS_2.0 µM**	$2.32x10^{15}$	36.92	32.1	30.0	40.6	1.15×10^{7}
**[+] InP/ZnS/_MC in PBS_4.2 µM**	$2.32x10^{15}$	36.92	58.2	62.3	34.95	2.83×10^{7}

J(λ) = Spectral overlap integral, R_0 = Förster distance, E^a = efficiency calculated from steady-state data, E^b = efficiency calculated from lifetime analysis, r = distance between the Donor and acceptor, $K_T(r)$ = rate of energy transfer.

[+] InP/ZnS QD decay analysis in the presence of [-] MC dye, at shorter time scale (50 ns):

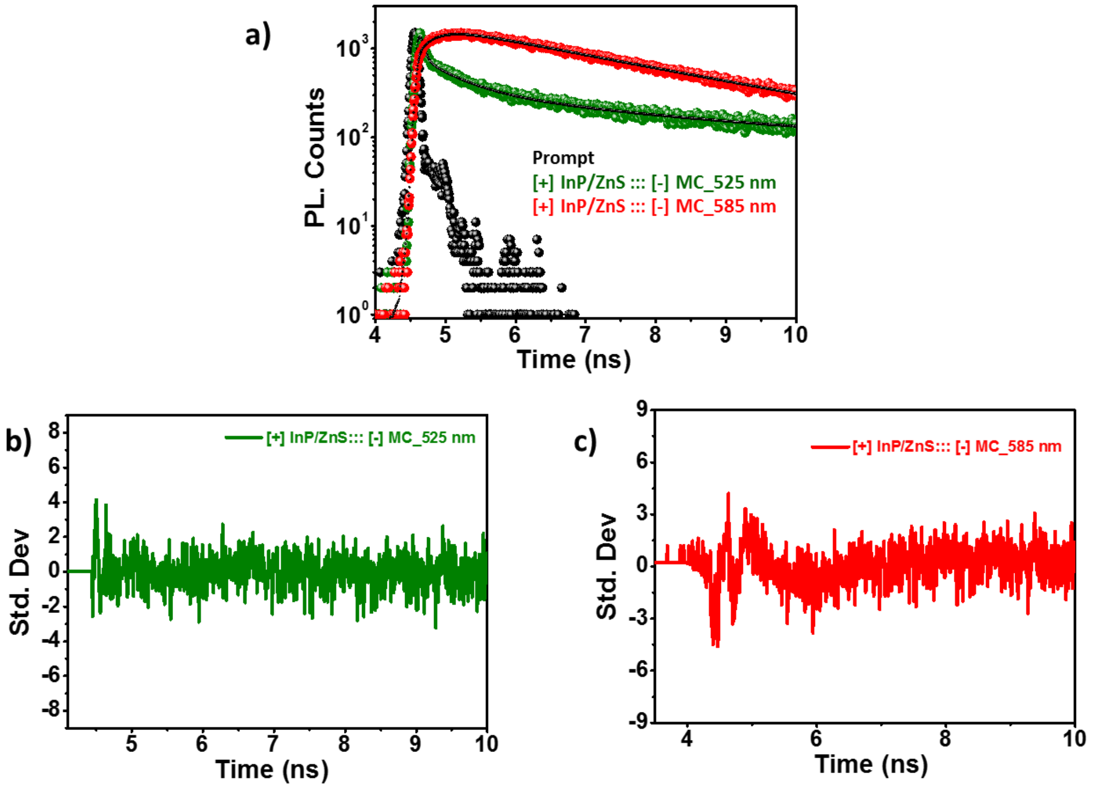

Fig. S13 Photoluminescence decay profile of [+] InP/ZnS::: [-] MC complex collected at the emission maxima of donor (525 nm) and acceptor (585 nm), at 50 ns time scale. The y-axis is plotted in log values. The same plot with y-axis in linear values is shown in Figure 3b in the main text. The residual plots of the fit corresponding to the decays collected at b) 525 nm and c) 585 nm is shown.

Table S4: Photoluminescence decay analysis of [+] InP/ZnS QD in the presence of [-] MC dye measured in a time window of 50 ns.

Sample	τ_1 (ns)	A_1	τ_2 (ns)	A_2	τ_3 (ns)	A_3	Chi.Sq
[+] InP/ZnS_MC_525 nm	2.58	0.15	0.53	0.79	24.2	0.05	1.16
[+] InP/ZnS_MC_585 nm	2.69	0.61	0.25	-0.36	12.8	0.03	1.14

Control experiments for electrostatically driven FRET:

Control experiment # 1

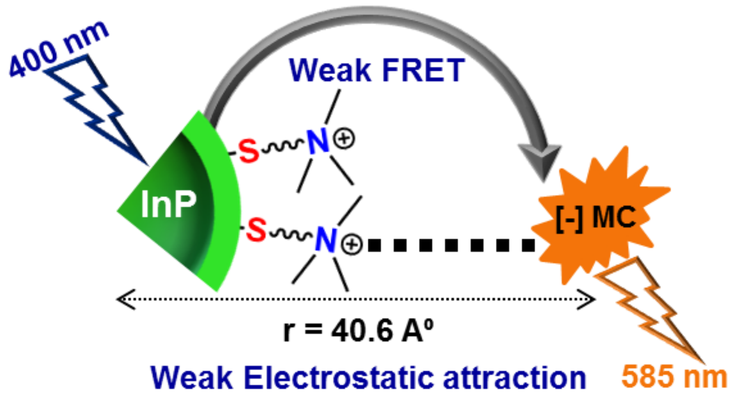

Scheme 1: Schematic representation of resonance energy transfer between [+] InP/ZnS QD and [-] MC dye under high salt conditions (PBS).

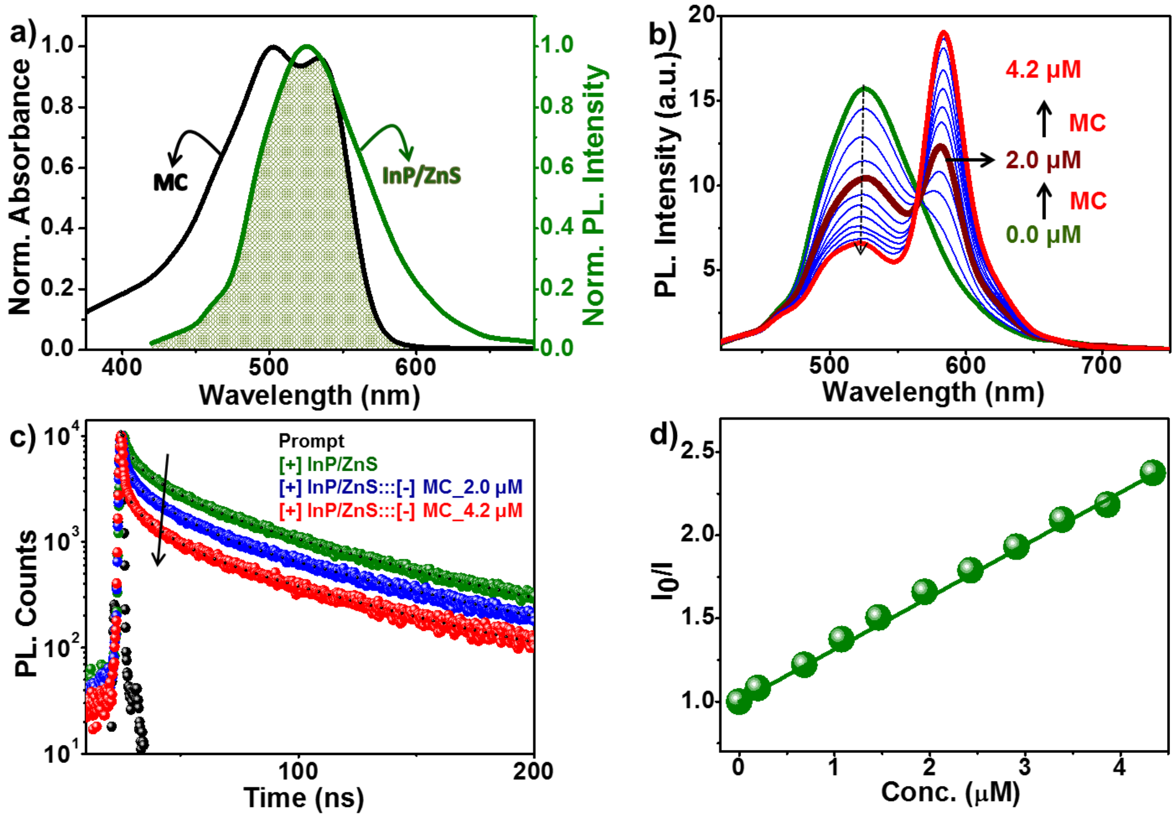

Fig. S14 a) Spectral overlap (shaded portion) between the photoluminescence spectrum of [+] InP/ZnS (green curve) in PBS and absorption spectrum of [-] MC (black curve). b) Spectral changes in the photoluminescence of [+] InP/ZnS QD on addition of [-] MC dye. c) Photoluminescence decay profile of [+] InP/ZnS in the absence (green decay) and presence of ~2.0 µM (blue decay) and 4.2 µM (red decay) of [-] MC, upon excitation with a 405nm laser source. d) Stern-Volmer plot showing the relative changes in the photoluminescence intensity of [+] InP/ZnS QDs as a function of MC dye concentration in PBS. Approximately twice the amount of the MC dyes (compared to water) was required to observe an energy transfer of ~ 60 % in [+] InP/ZnS::: [-] MC complex in PBS.

Control experiment # 2

Control experiments were carried out with [+] InP/ZnS QD as a donor and [+] CY dye as an acceptor. The charges are same on the surface of donor and acceptor, which prevented the complex formation, and thereby energy transfer.

a)

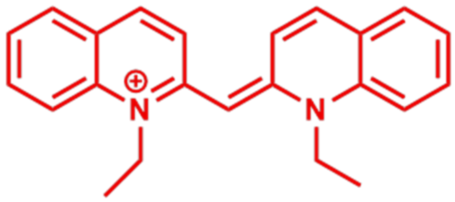

1, 1'-diethyl- 2, 2'-cyanine iodide (CY)

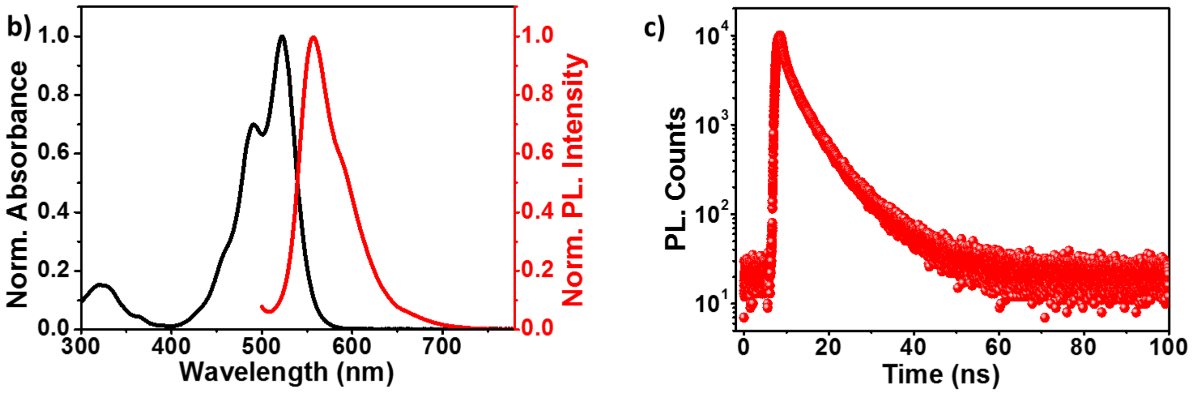

Fig. S15 a) Chemical structure of 1, 1’-diethyl- 2, 2'-cyanine iodide dye (CY). b) Normalized absorption (black curve) and photoluminescence (red curve) spectra of [+] CY dye in water. c) Photoluminescence decay profile of [+] CY dye in water upon excitation with a 405 nm laser source.

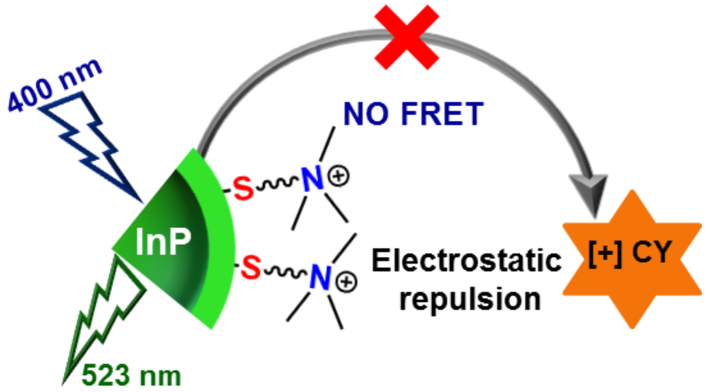

Scheme 2: Schematic representation of [+] InP/ZnS QD and [+] CY dye in water.

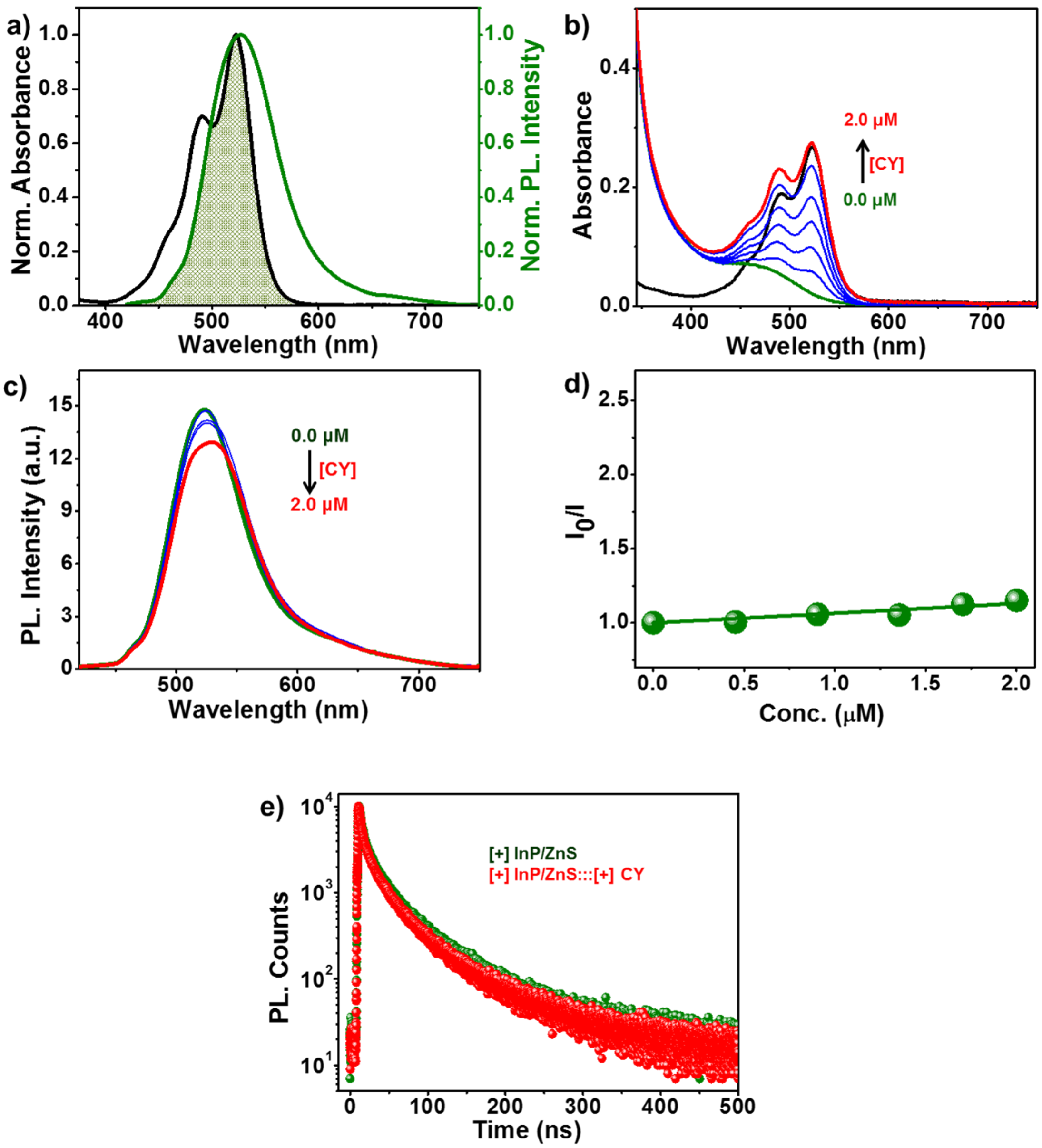

Fig. S16 a) Spectral overlap (shaded portion) between the photoluminescence spectrum of [+] InP/ZnS (green curve) and absorption spectrum of [+] CY (black curve). A large spectral overlap integral value of 1.56 x 10^{15} $M^{-1}cm^{-1}nm^{4}$ suggests that [+] InP/ZnS QDs and [+] CY dye form a good donor-acceptor pair. b) Absorption spectral changes of [+] InP/ZnS QDs with increasing concentration of [+] CY dye. The absorption spectrum of [+] CY alone is shown as the black curve. The absence of red shift in the absorption of dye in the presence of QDs rules out the formation of a strong ground state complex between [+] InP/ZnS QDs and [+] CY dye. c) Steady-state photoluminescence spectra of [+] InP/ZnS upon addition of varying concentrations of [+] CY dye. d) Stern-Volmer plot showing the negligible changes in the photoluminescence intensity of [+] InP/ZnS QDs as a function of CY dye concentration. e) Photoluminescence decay profile of [+] InP/ZnS in the absence (green decay) and presence (red decay) of 2.0 μM [+] CY dye, upon excitation with a 405 nm laser. All the steady state and time resolved experiments rules out the possibility of resonance energy transfer between [+] InP/ZnS QDs and [+] CY dye.

Control experiment # 3

Control experiments were carried out with [-] InP/ZnS QD as a donor and [-] MC dye as an acceptor. The charges are same on the surface of donor and acceptor, which prevented the complex formation, and thereby energy transfer.

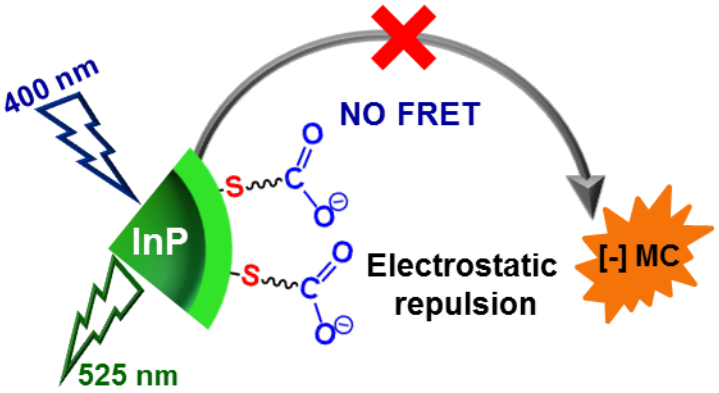

Scheme 3: Schematic representation of [-] InP/ZnS QD and [-] MC dye in water.

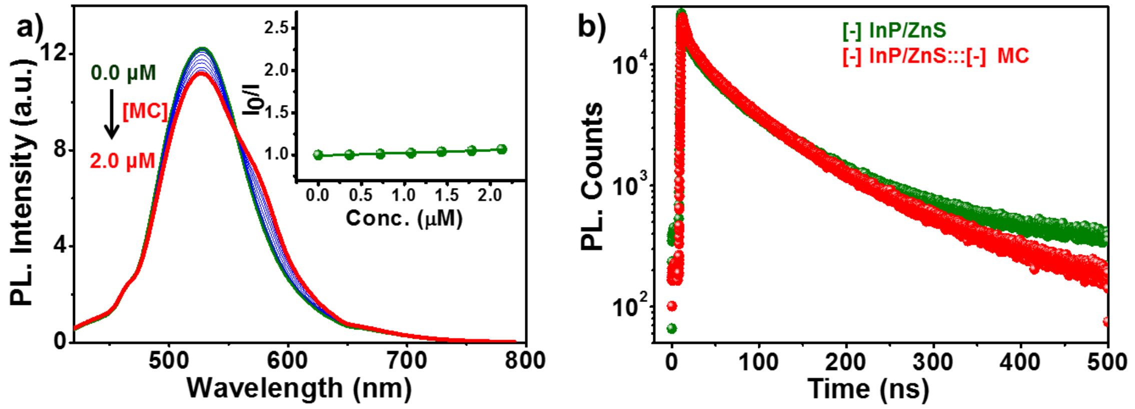

Fig. S17 a) Steady-state photoluminescence spectra of [-] InP/ZnS upon addition of varying concentrations of [-] MC. Inset shows the corresponding Stern-Volmer plot. b) Photoluminescence decay profile of [-] InP/ZnS in the absence (green decay) and presence (red decay) of [-] MC (2.0 μM), upon excitation with a 405 nm laser. All the steady state and time resolved experiments rules out the possibility of resonance energy transfer between [-] InP/ZnS QDs and [-] MC dyes.

Section 6: Stability of [+] InP/ZnS::: [-] MC complex

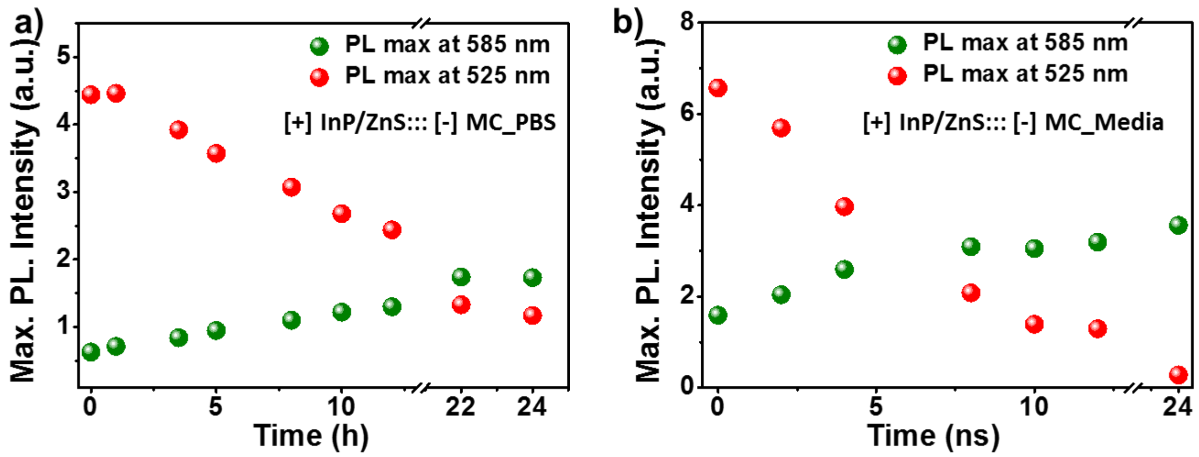

Fig. S18 The variation of photoluminescence intensity of [+] InP/ZnS::: [-] MC complex in a) PBS and b) DMEM media (with 10% FBS) monitored at donor (green dots) and acceptor emission (red dots) maxima. The [+] InP/ZnS::: [-] MC complex is dissociating in PBS and DMEM due to the breaking of electrostatic attraction by the ions present in the medium. The dissociation of the complex was accompanied with a reduction in the energy transfer process, and the emission of donor InP/ZnS QDs recovered with time.

References:

1 J. Tien, A. Terfort and G. M. Whitesides, *Langmuir* 1997, **13**, 5349–5355.

2 R. Xie, D. Battaglia and X. Peng, *J. Am. Chem. Soc.* 2007, **129**, 15432–15433.

3 A. Thomas, P. V. Nair and K. G. Thomas, *J. Phys. Chem. C* 2014, **118**, 3838–3845.

4 Z. A. Peng and X. Peng, *J. Am. Chem. Soc.* 2001, **123**, 183–184.

5 R. F. Kubin and A.N. Fletcher, *Chem. Phys. Lett* 1983, **99**, 49-52.

6 J. R. Lakowicz, Principles of Fluorescence Spectroscopy, 3rd ed., Springer: New York, **1999**.

7 P. Reiss, M. Protière, and L. Li, *Small* 2009, **5**, 154-168.
